# Photoacoustic Gas Sensing Using a Novel Fluidic Microphone Based on Thermal MEMS

**DOI:** 10.3390/s25247411

**Published:** 2025-12-05

**Authors:** Akash Gupta, Anant Bhardwaj, Achim Bittner, Alfons Dehé

**Affiliations:** 1 Hahn-Schickard, Wilhelm-Schickard-Str. 10, 78052 Villingen-Schwenningen, Germany; achim.bittner@hahn-schickard.de (A.B.); alfons.dehe@hahn-schickard.de (A.D.); 2 Ilmenau School of Green Electronics, Technical University Ilmenau, Ehrenbergstraße 29, 98693 Ilmenau, Germany; anant.bhardwaj@tu-ilmenau.de; 3 Georg H. Endress Chair of Smart Systems Integration, Department of Microsystems Engineering—IMTEK, Albert-Ludwigs-Universität Freiburg, Georges-Köhler-Allee 103, 79110 Freiburg, Germany

**Keywords:** photoacoustic, gas sensing, thermal sensor, microphone, non-resonant, acoustic sensor

## Abstract

Photoacoustic spectroscopy (PAS) is a powerful technique for selective gas detection; however, its performance in non-resonant configurations is fundamentally constrained by the poor low-frequency response of conventional acoustic detectors. Commercial MEMS microphones, although compact and cost effective, exhibit limited infrasound sensitivity, which restricts the development of truly miniaturised and broadband PAS systems. To address this limitation, we present a novel MEMS fluidic microphone (f-mic) that operates on a thermal sensing principle and is explicitly optimised for the infrasound regime. The sensor demonstrates a constant sensitivity of 32 μV/Pa for frequencies below 20 Hz. A detailed analytical model incorporating frequency-dependent effects is developed to identify and investigate the critical design parameters that influence system performance. The overall system exhibits a band-pass frequency response, enabling broadband operation. Based on these insights, a miniaturised photoacoustic cell is fabricated, ensuring efficient optical coupling and f-mic integration. Experimental validation using a CO_2_-targeted laser system demonstrates a linear response up to 5000 ppm, a sensitivity of 6 nV/ppm, and a theoretical detection limit of 300 ppb over 100 s, resulting in an NNEA of $6\times 10^{-6}$ W cm^−1^ Hz^−0.5^. These results establish the f-mic as a robust, scalable solution for non-resonant PAS, effectively overcoming a significant bottleneck in compact gas sensing technologies.

## 1. Introduction

Photoacoustic spectroscopy (PAS) is a gas detection technique that exploits the photoacoustic effect, in which modulated light is absorbed by target gas molecules and converted into heat. The resulting periodic thermal expansion generates acoustic pressure within a closed volume, which can be detected using an acoustic transducer such as a microphone. In the weak absorption limit, the generated photoacoustic pressure is proportional to the gas concentration [1]. This technique enables direct and selective detection without requiring long optical paths or bulky spectral instruments. Unlike long-path optical techniques, such as non-dispersive infrared spectroscopy (NDIR) and tunable laser spectroscopy (TLS), PAS sensitivity is primarily governed by the available optical power and the efficiency of acoustic transduction, rather than the optical path length, allowing for compact, miniaturised gas sensors with high sensitivity [2]. The combination of spectral selectivity from tunable lasers and compactness has made PAS a highly suitable technology for trace-gas detection in applications such as environmental monitoring, automotive emissions, medical and health diagnostics, and industrial process control and safety.

The choice of acoustic detector determines the operational mode of a PAS system, which can be either resonant or non-resonant, and it significantly influences performance metrics, including bandwidth, dynamic range, and robustness. Considerable research has focused on resonant detection schemes, such as quartz tuning forks (QEPAS) [3] and cantilevers (CEPAS) [4,5,6], which provide high sensitivity by exploiting mechanical resonance. Other approaches include piezoelectric resonators and micro resonators, which similarly rely on resonance. While such detectors achieve sensitivities in the ppb range, they are inherently limited by narrow bandwidths, fragile structures, environmental disturbances, and complex readout requirements, posing a real challenge to cost-effective commercialisation [7,8]. To address these challenges, complex algorithms are needed for reliable, robust detection [9]. In contrast, non-resonant systems offer broader frequency response, improved robustness, and reduced sensitivity to imperfections, making them more suitable for miniaturised and commercial devices. Commercial MEMS microphones have been widely adopted in this context due to their broadband response, small size, and low cost [10,11]. However, their low-frequency (a few tens of Hz) performance remains limited, constraining their application in non-resonant PAS.

Non-resonant PAS requires high optical power sources to compensate for the absence of acoustic resonance. While lasers offer high modulation depth and stable output, most compact systems rely on thermal emitters, which behave as blackbody sources and are limited by their modulation speed. Commercial MEMS microphones, typically optimised for the audio range (20 Hz–20 kHz), deliver good broadband sensitivity but are restricted at low frequencies [12,13,14,15], which are particularly relevant for non-resonant PAS. Their performance is further constrained by mechanical damping mechanisms, such as squeeze-film damping [16], which significantly contributes to the overall noise. Since non-resonant PAS relies on the direct conversion of optical energy into acoustic pressure, these limitations restrict the achievable performance in the desired frequency range. Alternative detector technologies that are inherently robust and more sensitive in the low-frequency regime are therefore of significant interest. A qualitative comparison of the different detectors used for Photoacoustic sensing, in terms of detection type, sensitivity, size, and bandwidth, is provided in Table 1.

Thermal sensors, such as calorimetric and thermal flow sensors, provide a promising alternative for low-frequency acoustic detection. Thermal flow sensors transduce pressure-driven flow or particle velocity into a temperature signal using highly sensitive thermal elements, and can be fabricated via MEMS processes with no moving parts, providing enhanced robustness [17]. Their sensitivity can be tailored through surface micro-machining, enabling favourable broadband and low-frequency performance as demonstrated in particle velocity sensing applications [18,19,20]. To address the limitations of MEMS microphones in non-resonant PAS, we previously developed a MEMS fluidic microphone based entirely on thermal transduction [21]. This device exhibited a flat infrasound response, indicating its potential as a robust, miniaturised acoustic sensor for PAS. Building on this foundation, the present work validates the feasibility of integrating this sensor into a non-resonant PAS system for gas detection.

CO_2_ is used as a test gas due to its availability, ease of handling, and non-toxicity. We utilise a laser-based setup due to its multiple advantages over other thermal IR emitters. To achieve the goal of investigating detector performance, a laser provides high power and high modulation depth. This allows for a systematic and more accurate characterisation of the detector in a PAS system. Firstly, we present the operating principle and acoustic characterisation results for the fluidic microphone. Given its limited frequency bandwidth and superior performance at low frequencies, we opt for a non-resonant PAS configuration. Secondly, we compare the PAS concept when integrated with a commercial microphone versus a fluidic microphone. Then, we develop a detailed analytical model that accounts for all frequency-dependent effects crucial to system design and optimisation. We also outline the key considerations necessary for designing a detector cell to achieve optimal sensor integration. A detailed experimental investigation of the system’s performance is presented, focusing on the dependence on various design and physical parameters, including the frequency response and cell volume. Finally, we discuss the system’s long-term stability, highlighting its performance limits, and conclude with a summary of our findings and future research directions.

## 2. Theory

### 2.1. The Fluidic Microphone as PA Detector

**Figure 1:**
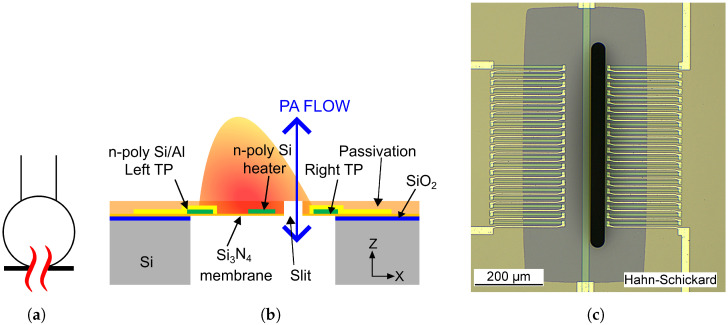
(**a**) Electrical symbol, (**b**) descriptive cross-section and (**c**) microscopic top view of the fluidic microphone. The PA flow through the slit generates a convective cooling at the right thermopile (TPR), leading to an oscillatory voltage signal.

The fluidic microphone, used as an acoustic detector, differs from the traditional capacitive microphone in several ways. The traditional microphone directly measures the PA pressure and converts it into a voltage signal. However, the fluidic microphone relies on the generation of an acoustic flux due to the PA pressure. This flux ultimately creates temperature oscillations through convective heat transfer, which are then converted to voltage due to the Seebeck effect at the right thermopile (TPR). The detailed fabrication process and the chip dimensions can be found in [21,22]. The electrical symbol with the cross-sectional view indicating all the components of the fluidic microphone is displayed in Figure 1. The top-view image of the chip design used in this work is also shown. The left thermopile (TPL) serves as a passive component and will not be used in this work.

**Figure 2:**
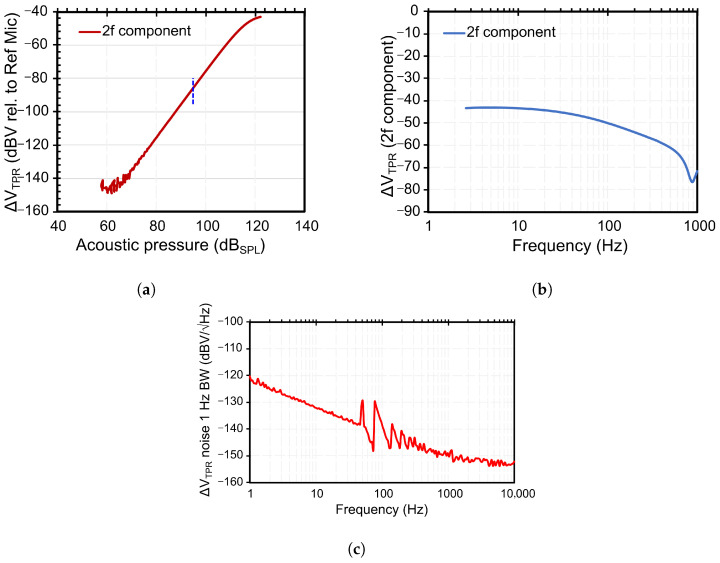
Acoustic characterisation results and noise sweep of the fluidic microphone. (**a**) The pressure response measured at a heater current of 2 mA and an acoustic frequency of 20 Hz ($f_{\mathrm{ak}}$), (**b**) frequency sweep shows a low-pass filter response with constant sensitivity up to 20 Hz, and (**c**) spectral noise distribution shows the flicker noise dominating up to 1 kHz frequency.

The sensor is characterised acoustically using the measurement setup published previously [21,22] to extract the sensitivity and the frequency response. The results are shown in Figure 2. For a heater current of 2 mA, a sensitivity of −90 dBV/Pa (or 32 µV/Pa) is achieved. More importantly, the sensor shows a flat frequency response below 20 Hz, unlike a membrane-based capacitive microphone. Noise measurements show a strong 1/f noise behaviour as expected due to the thermal components. For a 20 Hz acoustic signal, the noise is −140 dB V/$\sqrt{\mathrm{Hz}}$, which converts into an SNR of approximately 50 dB. The sensitivity dependence on the acoustic pressure $p_{\mathrm{ak}}$ and the heater temperature $T_{h}$ can be expressed as

(1) $$S_{f}\propto p_{\mathrm{ak}}^{2}\cdot T_{h}.$$

As compared to a traditional MEMS microphone, the fluidic microphone, being a thermal sensor, offers some drawbacks such as higher power consumption and influence of the environmental conditions such as temperature variations.

#### Acoustic vs. Photoacoustic Pressure Detection

In conventional acoustics, pressure fluctuations result from longitudinal waves, creating symmetric deviations around equilibrium pressure. For instance, a loudspeaker produces a sinusoidal pressure wave through mechanical displacement, leading to an oscillatory flow with a zero time-average. In contrast, photoacoustic pressure in gases comes from the absorption of modulated optical radiation, which causes localised heating and thermal expansion. This process creates an asymmetric waveform, typically featuring a sharp rise during illumination and a slower thermal relaxation, resulting in primarily positive excursions with a non-zero time-average. The fluidic microphone responds differently to this distinction. For an acoustic pressure, the output signal is generated at double the acoustic frequency given by $∆V_{\mathrm{TPR}}\left(2f_{\mathrm{ak}}\right)$. In contrast, for PA pressure, the output is detected as a thermopile voltage at the infrared modulation frequency expressed as $∆V_{\mathrm{TPR}}\left(f_{\mathrm{mod}}\right)$. This explains the quadratic dependence of its sensitivity on the acoustic pressure as mentioned above in Equation (1).

**Figure 3:**
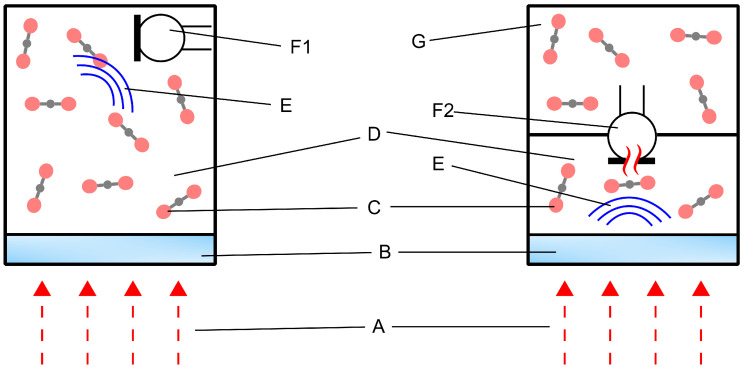
Photoacoustic pressure detection concept using a conventional microphone (**left**) vs. a fluidic microphone (**right**). The description is as follows: A = Modulated infrared radiation, B = Optical window, C = Target gas (CO_2_), D = Active (front) volume, E = PA pressure, F1 = MEMS microphone, F2 = MEMS fluidic microphone (this work) and G = Passive (back) volume.

In Figure 3, a comparison of the integration of the fluidic microphone and a commercial microphone in a PA detector cell is shown. The commercial microphone can be directly sealed inside a small volume consisting of the target gas. On the other hand, the fluidic microphone, which relies on flow detection, requires two volumes: the active volume and the passive volume. The PA pressure is generated in the active volume, which generates a flow through the micro-perforation to the passive volume. In this way, the integration of these two sensors inside a detector cell varies. More importantly, the fluidic microphone is a thermal sensor, which means that its direct interaction with the incident IR radiation must be avoided to prevent additional thermal noise. This is not a significant issue with a commercial microphone.

### 2.2. Model and Simulation

**Figure 4:**
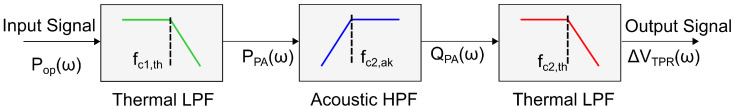
Block diagram for the complete frequency chain of the fluid microphone integrated PAS detector cell.

In this section, an analytical model is developed that incorporates all the critical frequency-dependent effects related to the fluidic microphone-based PAS. This enables us to understand the frequency behaviour of the system and investigate the various physical parameters that affect system performance. Being a multi-physical coupled system, three significant frequency-related effects in the thermal and acoustic domains are modelled to simulate the overall system behaviour. The signal chain consisting of these frequency effects is shown in Figure 4. From the frequency modulated input optical power $P_{\mathrm{op}}\left(\omega \right)$ to the output PA signal measured at the thermopile $∆V_{\mathrm{TPR}}$, all the effects are discussed in detail.

#### 2.2.1. PA Pressure Generation

The input power modulated at a frequency ω (=2πf) reaches the front volume $V_{\mathrm{fv}}$ (in m^3^) of the detector cell containing the target gas. The power is absorbed, resulting in the generation of pressure due to the photoacoustic effect widely expressed as [23]

(2) $$P_{\mathrm{PA}}\left(\omega \right)=\frac{j\left(\gamma -1\right)P_{\mathrm{abs}}}{\omega V_{\mathrm{fv}}\left(1+\frac{j}{\omega \tau_{\mathrm{th}}}\right)}.$$

γ,n,α are the adiabatic constant, mole fraction, and the absorption coefficient (in cm^−1^) of the gas, respectively. $P_{\mathrm{abs}}$ is the absorbed IR power inside the PA cell and is expressed using Beer–Lambert’s law [24]. This can be used to derive the amplitude of the PA pressure as

(3) $$|P_{\mathrm{PA}}\left(\omega \right)|=\frac{\left(\gamma -1\right)\left(1-e^{-n\alpha l_{\mathrm{abs}}}\right)P_{\mathrm{op}}\cdot \tau_{\mathrm{th}}}{V_{\mathrm{fv}}\sqrt{1+{\left(\omega \tau_{\mathrm{th}}\right)}^{2}}},$$

where $P_{\mathrm{op}}$ is the optical power (in W) reaching the detector cell. $\tau_{\mathrm{th}}$ (s) represents the time constant for the thermal losses involved in the pressure generation. These losses depend on the difference in thermal conductivities between the gas and the detector cell, as well as the thermal mass of the sealed gas volume. This conversion process behaves like a damped first-order low-pass electrical filter, implying that below a certain frequency $f<1/\tau_{\mathrm{th}}$, gas has sufficient time to heat, and hence, the PA pressure generation is the highest. As derived in [6], $\tau_{\mathrm{th}}$ increases quadratically with the radius of the detector cell $r_{\mathrm{fv}}$ and can be expressed as

(4) $$\tau_{\mathrm{th}}=r_{\mathrm{fv}}^{2}/\zeta ,$$

where ζ is a constant depending on the geometry of the cell and thermal properties of the gas. It will be experimentally extracted in this work. Finally, Equation (3) can be rewritten as

(5) $$\begin{array}{c} |P_{\mathrm{PA}}\left(\omega \right)|=P_{0}\cdot A_{1}\left(\omega \right), \end{array}$$

(6) $$\begin{array}{c} A_{1}\left(\omega \right)=\frac{\tau_{\mathrm{th}}}{\sqrt{1+{\left(\omega \tau_{\mathrm{th}}\right)}^{2}}}\;\mathrm{and}\;P_{0}=\frac{\left(\gamma -1\right)\left(1-e^{-n\alpha l_{\mathrm{abs}}}\right)P_{\mathrm{op}}}{V_{\mathrm{fv}}}. \end{array}$$

#### 2.2.2. Acoustic-Flux Generation

This effect is specific to the fluidic microphone and is one of the most significant factors in determining its sensitivity. The generated PA pressure in the front volume creates a pressure difference across the perforation in the membrane. This leads to an oscillating volume flow (or flux) through the perforation, which generates a convective cooling effect at the TPR and the heater at the frequency of IR modulation. The goal is to maximise this flux for a certain PA pressure to achieve high sensitivity.

In terms of a mechanical analogy, the flux behaviour is comparable to an oscillating mass connected between two hinged springs. In the acoustic domain, this corresponds to the mass of the air oscillating between the acoustic compliances of the front and back volumes. The effective compliance of such a system can be expressed as the parallel combination of the front volume and the back volume compliance as

(7) $$\begin{array}{c} \frac{1}{C_{\mathrm{eff}}}=\frac{1}{C_{\mathrm{fv}}}+\frac{1}{C_{\mathrm{bv}}}. \end{array}$$

This can be further expressed in terms of volume by using the compliance from the literature as $C=V/\rho c^{2}$ [25] (ρ is the density and *c* is the speed of sound in the sealed gas mixture)

(8) $$\frac{1}{V_{\mathrm{eff}}}=\frac{1}{V_{\mathrm{fv}}}+\frac{1}{V_{\mathrm{bv}}}.$$

The flux is calculated using the total acoustic impedance of the system, given as

(9) $$\begin{array}{c} Z_{\mathrm{ak}}\left(\omega \right)=R_{\mathrm{perf}}+\frac{1}{j\omega C_{\mathrm{eff}}},\;\mathrm{or} \end{array}$$

(10) $$\begin{array}{c} |Z_{\mathrm{ak}}\left(\omega \right)|=\sqrt{R_{\mathrm{perf}}^{2}+{\left(\frac{1}{\omega C_{\mathrm{eff}}}\right)}^{2}}. \end{array}$$

$R_{\mathrm{perf}}$ is the viscous resistance offered by the perforation. Now, the magnitude of the flux can be written as

(11) $$\begin{array}{cc} \left|Q(\omega )\right| & =|\frac{P_{\mathrm{PA}}\left(\omega \right)}{Z_{\mathrm{ak}}\left(\omega \right)}|. \end{array}$$

Now, from Equations (9) and (5), and using the values of the $C_{\mathrm{eff}}$ and the $R_{\mathrm{perf}}$ from [25], the flux velocity is expressed as

(12) $$\left|v\left(\omega \right)\right|=\frac{\left|Q(\omega )\right|}{A_{\mathrm{perf}}}=\frac{P_{0}}{A_{\mathrm{perf}}}\cdot A_{1}\left(\omega \right)\cdot A_{2}\left(\omega \right),$$

where

(13) $$\begin{array}{c} A_{2}\left(\omega \right)=\frac{\omega C_{\mathrm{eff}}}{\sqrt{1+{\left(\omega \tau_{\mathrm{leak}}\right)}^{2}}}\;\mathrm{and}\;\tau_{\mathrm{leak}}=R_{\mathrm{perf}}\cdot C_{\mathrm{eff}}. \end{array}$$

This means that $A_{\mathrm{perf}}$ and $C_{\mathrm{eff}}$ are two of the most critical design parameters for the fluidic microphone, determining the dynamic response and sensitivity of the PA system. The output PA signal and $\tau_{\mathrm{leak}}$ depend linearly with $V_{\mathrm{eff}}$ and inversely with the $A_{\mathrm{perf}}$.

#### 2.2.3. Thermopile Detection

**Figure 5:**
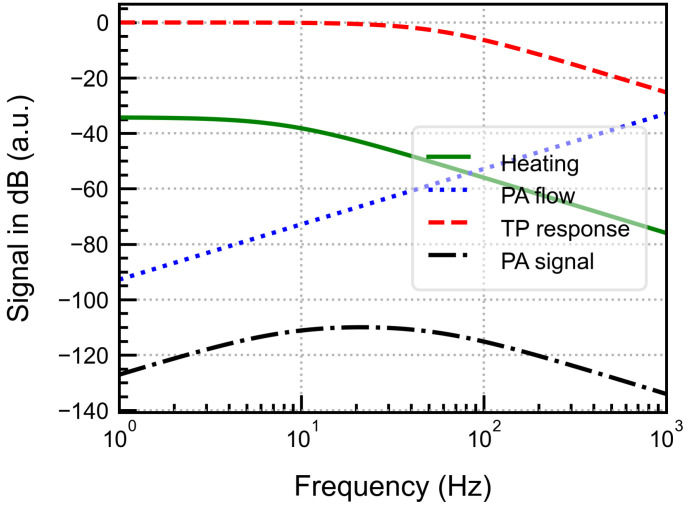
Simulated frequency effects involved in the fluidic microphone PAS detection.

The generated flux cools the hot contact of the TPR through forced convection, creating an oscillation in the heat flux. These oscillations are converted into temperature oscillations, which can be eventually read out as a differential Seebeck voltage. This is the final output signal, also known as the photoacoustic signal. However, due to the thermal mass of the fluidic microphone, the conversion from heat to temperature behaves like a low-pass filter. The corresponding time constant $\tau_{\mathrm{TP}}$ depends on the thermal mass of the microphone, which can be expressed using a transfer function of a first-order low-pass filter [26] as

(14) $$\begin{array}{cc} A_{3}\left(\omega \right) & =\frac{T_{h}\cdot n_{\mathrm{TE}}}{\sqrt{1+{\left(\omega \tau_{\mathrm{TP}}\right)}^{2}}}. \end{array}$$

where $T_{h},n_{\mathrm{TE}}$ are the operating temperature of the heater and the number of thermo-elements contributing to the Seebeck voltage (20 in this work). On combining all the frequency effects, the PA signal is expressed as

(15) $$\begin{array}{c} ∆V_{\mathrm{TPR}}\left(\omega \right)=∆V_{\mathrm{PA}}\left(\omega \right)=\frac{P_{0}}{A_{\mathrm{perf}}}\cdot A_{1}\left(\omega \right)\cdot A_{2}\left(\omega \right)\cdot A_{3}\left(\omega \right) \end{array}$$

(16) $$\begin{array}{c} |∆V_{\mathrm{PA}}\left(\omega \right)|=P_{0}\left(\frac{\tau_{\mathrm{th}}}{\sqrt{1+{\left(\omega \tau_{\mathrm{th}}\right)}^{2}}}\right)\left(\frac{\omega C_{\mathrm{eff}}}{A_{\mathrm{perf}}\cdot \sqrt{1+{\left(\omega \tau_{\mathrm{leak}}\right)}^{2}}}\right)\left(\frac{T_{h}\cdot n_{\mathrm{TE}}}{\sqrt{1+{\left(\omega \tau_{\mathrm{TP}}\right)}^{2}}}\right) \end{array}$$

where $\zeta ,C_{\mathrm{eff}}$, and $G=P_{0}T_{h}n_{\mathrm{TE}}/A_{\mathrm{perf}}$ are used as the fitting parameters. From Equation (16), the dependence of the photoacoustic signal on different system and design parameters of the detector cell and the fluidic microphone can be derived. The different frequency effects, using the parameters listed in Table 2 are plotted in Figure 5.

The simulation results show that the output PA signal exhibits broadband behaviour where the thermal leakage cut-off frequency determines the lower limit, and the thermal mass of the chip determines the upper limit. However, the signal amplitude at a constant frequency depends on the perforation area and the effective volume. These parameters can be used to control the frequency response for the desired operational bandwidth, providing flexibility in tuning system performance.

## 3. Materials and Methods

### 3.1. CO_2_ IR Absorption and Laser Selection

**Figure 6:**
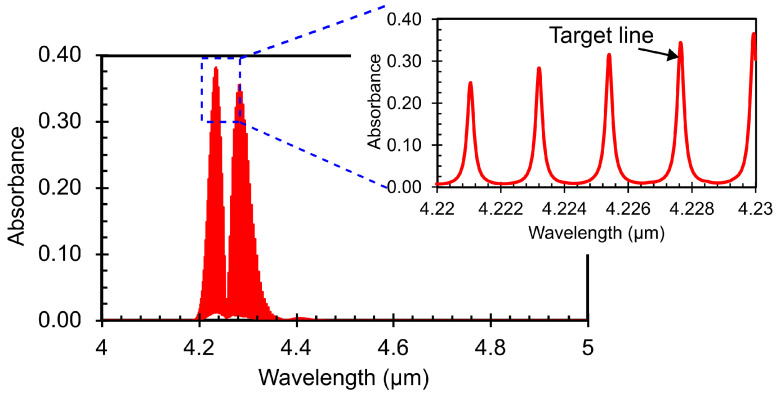
IR absorption lines for 1000 ppm CO_2_ at 295 K, 1 atm simulated using the HITRAN database [27].

**Figure 7:**
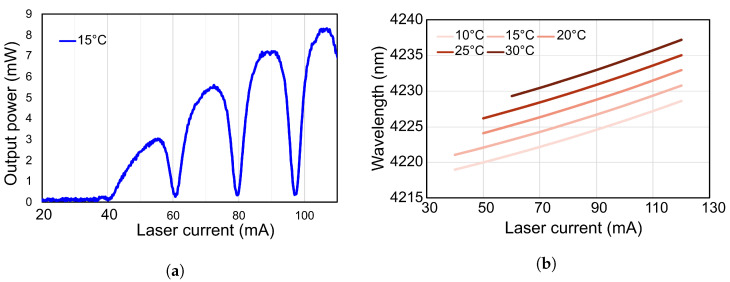
(**a**) DFB laser output power at 15 °C and (**b**) emitted wavelength dependence on operating current at different temperatures.

CO_2_ gas strongly absorbs IR in the 4–4.4 μm wavelength range. The simulated IR absorption lines for CO_2_ gas at 295 K and 1 atm are shown in Figure 6. The data was taken from the HITRAN database [27]. The selection of the optical excitation source is a critical factor in PA gas sensing. Therefore, to target this strongly absorbing region, a specially optimised Distributed Feedback (DFB) laser from Nanoplus GmbH (Meiningen, Germany) is used as the IR source. The laser is integrated into a housing that includes a collimator and a thermo-electric cooler (TEC) for temperature control. The emission characteristics of the laser, such as the emitted wavelength $\lambda_{\mathrm{em}}$ and the output power $P_{\mathrm{op}}$, are both dependent on the supplied current. This dependence is shown in Figure 7. The output power of the laser as well as the wavelength increase with the input current. The drop in the laser power is due to the presence of CO_2_ in the atmosphere during its characterisation as confirmed by the manufacturer. Hence, to achieve optimal performance and maximum sensitivity, we operate the laser using a current that targets the IR line near 4228 nm. The operating current will be extracted experimentally, as it can depend on several external factors such as the geometry of the PA cell and the measurement conditions.

### 3.2. Detector Cell

**Figure 8:**
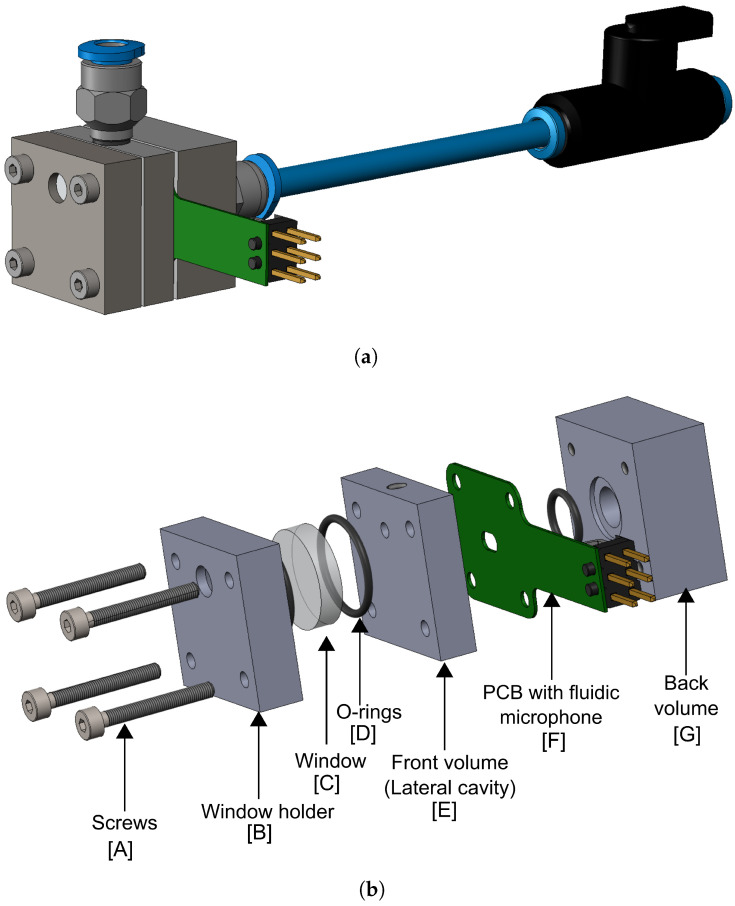
(**a**) The 3D model, and (**b**) the exploded view with all the components of the detector cell designed for the photoacoustic measurements.

**Figure 9:**
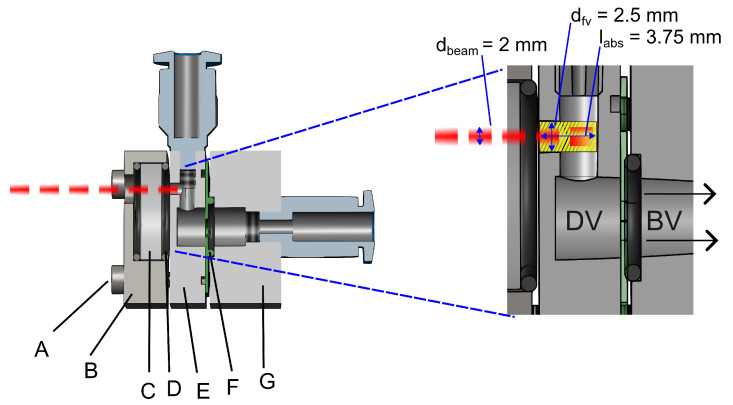
Cross-section and inner dimensions of the detector cell. The green area is the front (active) volume; DV is the dead volume contributing to thermal leakage; and BV is the back (passive) volume. The red line is the modulated laser beam.

The design of the detector cell, integrated with the fluidic microphone, is the main focus of this work. Various design factors and limitations were considered to optimise the performance of the PAS system. The main challenge was the direct interaction of the laser beam with the fluidic microphone. As a thermal sensor, it can absorb IR radiation, resulting in high thermal noise levels, eventually reducing its sensitivity to detect low gas concentrations. To avoid this, we used a lateral cavity approach as shown in Figure 8. The cross-sectional view of the detector cell, marked with all relevant dimensions, is shown in Figure 9. The front volume $V_{\mathrm{fv}}$ is a cylinder with diameter $d_{\mathrm{fv}}=2.5$ mm and $l_{\mathrm{abs}}=3.75$ mm. The dimensions are chosen to maintain a small front volume while coupling the laser beam with a $d_{\mathrm{beam}}=2$ mm diameter inside the cell through an optical window. The window (WG30530, Thorlabs GmbH, Bergkirchen, Germany) has a 12.7 mm diameter and 5 mm thickness, and shows around 90% IR transmission for the target CO_2_ absorption target. There is some additional volume referred to as the dead volume $V_{d}$. As explained via the model, the fluidic microphone requires a well-defined back volume that facilitates the transmission of the photoacoustic flow through the perforation, thereby generating a maximum convective cooling effect and leading to a high output signal at the TPR. Therefore, a combination of tubes and valves is used to vary the back volume in experiments easily. The gas mixture is allowed to flow through the perforation on the sensor and manually sealed for gas concentration measurements.

### 3.3. Measurement Setup

**Figure 10:**
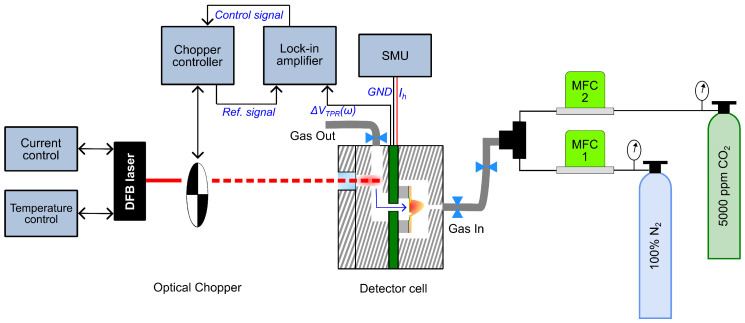
Block diagram of the PAS measurement setup with the cross-section view of the detector cell.

The entire measurement setup is shown in Figure 10. The laser is operated using a constant current under controlled temperature conditions. This is achieved using a laser current driver (KLD101) and a temperature controller (TTC001) from Thorlabs GmbH. An optical chopper is used to modulate the frequency of the collimated laser beam mechanically in the range of 3 Hz to 3 kHz. The detector cell and the chopper are mechanically and optically aligned with the laser beam on an optical bench with the help of an Infrared detection card. A lock-in amplifier from Zurich Instruments AG is used to control the chopper frequency and simultaneously to measure the photoacoustic signal from the thermopile. The component of the TPR voltage at the chopper frequency is demodulated. The heater on the fluidic microphone operates in constant current mode at 2.5 mA using a source and measurement unit (SMU) from Keithley Instruments (Cleveland, OH, USA).

The gas mixing setup consists of two mass flow controllers (SFC5500) from Sensirion AG (Stäfa, Switzerland), which are calibrated for 100% N_2_. Then, 5000 ppm of CO_2_ is diluted with pure N_2_ while keeping the total flow rate constant to 50 mL/min (cm^3^/min). A low flow rate is crucial for gas filling inside the detector cell to prevent any damage that can be caused to the MEMS sensor due to the gas flowing through the chip membrane. The target gas flows from the inlet through the chip to the outlet, thoroughly flushing the system and establishing a constant concentration inside the cell. Once the desired concentration is reached, the flow is stopped before data acquisition by manually closing the valves. Special care is taken to avoid overpressure in the cell by first closing the inlet valve and then the outlet valve. Thus, during the actual photoacoustic measurement, the gas is static (no flow), preventing flow-induced disturbances that could interfere with detection.

The present measurements were performed with gas supplied from cylinders under relatively constant laboratory conditions, thereby minimising short-term variations in gas and ambient temperatures. Nevertheless, both gas and ambient temperature can influence the sensor response.

## 4. Results and Discussion

### 4.1. Laser Emission Line Sweep

**Figure 11:**
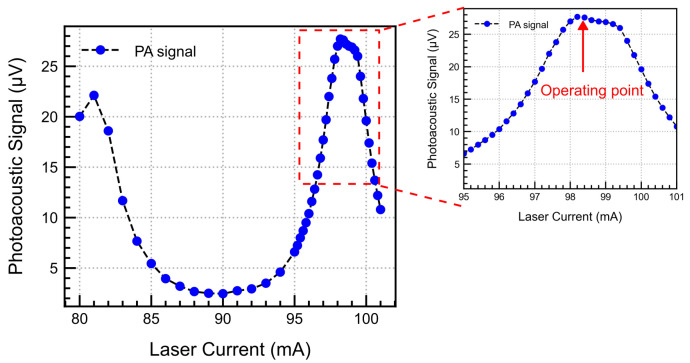
Dependence of the PA signal on the laser current for 5000 ppm CO_2_ at 40 Hz, 0.5 mL back volume.

This measurement was performed to determine the optimal operating current for the laser, where the laser emission and CO_2_ absorption lines align, thereby achieving the maximum possible sensitivity and minimising the impact from other interfering gases. We swept the current from 80 to 101 mA in steps of 1 mA, at a constant temperature of 15 °C. The detector cell was sealed with 5000 ppm CO_2_, and the TP signal was continuously recorded at a chopper frequency of 40 Hz. The results are plotted in Figure 11. As expected, the PAS signal increases when the laser matches the CO_2_ absorption lines. The first peak near 80 mA is slightly lower than the second peak near 100 mA. This is due to the difference in the line intensities as shown in Figure 6. For maximum sensitivity, we chose 98.2 mA as the laser current for all further measurements.

### 4.2. Optimal Modulation Frequency

The frequency response of the PA signal was measured by varying the chopper frequency from 2 to 200 Hz and continuously recording the PA signal. This was performed to validate the simulated frequency behaviour and to determine the frequency at which the maximum signal is obtained, thereby achieving the maximum possible SNR. The measurements were performed at 5000 ppm CO_2_, a back volume of 0.5 mL, and a laser current of 98.2 mA. The measurements were repeated three times under similar environmental conditions to verify the accuracy and reproducibility of the results, as well as the error introduced by the manual gas sealing process.

**Figure 12:**
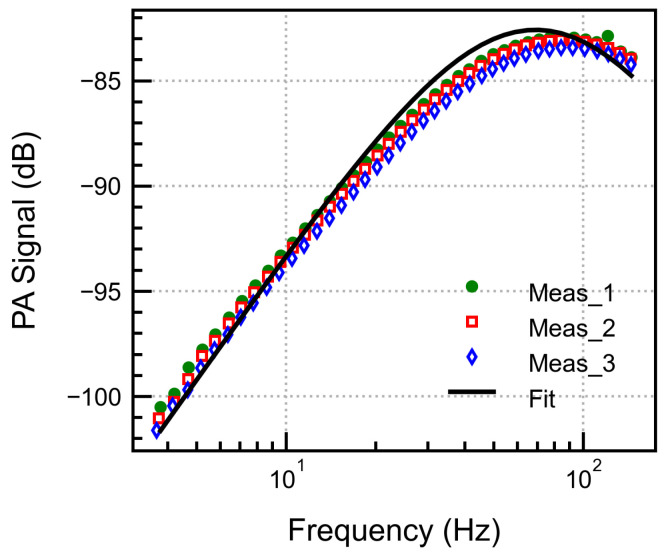
Frequency dependence of the PA signal measured three times and fitted to the frequency response analytical model.

As seen from the measurement results in Figure 12, the curves overlap, with a peak close to 70 Hz indicating good repeatability (−85.23 dB ± 0.15 dB for n = 3) and reliability of the experimental data. Equation (16) was used to fit the measured data with fit parameters as ζ=6.3, $C_{\mathrm{eff}}=9.4\times 10^{-13}$ m^3^/Pa, G=5. The increased value of $C_{\mathrm{eff}}$ signifies the underestimation in the calculated front volume by neglecting the gas volume in the valve and the non-illuminated dead volume. The value of ζ signifies a more substantial thermal leakage effect due to the uneven geometry and operation in the boundary layers.

### 4.3. Back Volume Dependence

**Figure 13:**
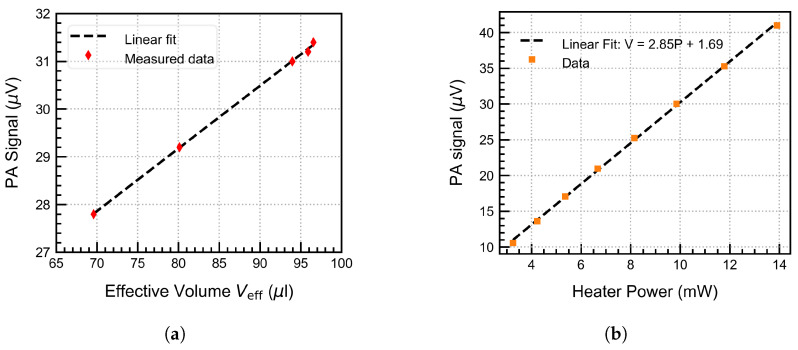
(**a**) PA signal dependence on the effective volume, and (**b**) the heater electrical power (units for the fit parameters are the same as the plotted data units).

The dependence of the PA signal on the effective volume was validated experimentally. This was achieved by varying the back volume to four different values from BV1 = 0.25 cm^3^ (250 μL), BV2 = 0.47 cm^3^, BV3 = 8.64 cm^3^, BV4 = 53.47 cm^3^, which indirectly increased the effective volume (refer to Equation (8)). The PA signal was measured and averaged over 200 s for each point while keeping the other parameters constant. The results, plotted in Figure 13a, show a linear dependence of the PA signal with $V_{\mathrm{eff}}$ as expected from Equation (8). The PA signal and the effective volume increase linearly at first, then saturate at high back volume. This suggests that the front volume will limit the performance of the PA system; hence, an appropriately sized back volume would yield the highest signal with the smallest possible system size. These results highlight the importance of the volume design in a fluidic microphone integrated PAS cell.

### 4.4. Heater Power Sweep

The heater power was varied from 3 to 14 mW by increasing the heater current from 0.5 to 3 mA. This increased the heater operating temperature from approximately 40 °C up to 200 °C. The PA signal was continuously recorded at all operating conditions and parameters, which were held constant throughout the measurement. The results are plotted in Figure 13b. We observed a linear increase in the output voltage because the sensitivity of the fluidic microphone increased linearly within this temperature range, thus increasing the PA signal as also expected from the theoretical model (ref. Equation (16)).

### 4.5. Gas Concentration Measurements

**Figure 14:**
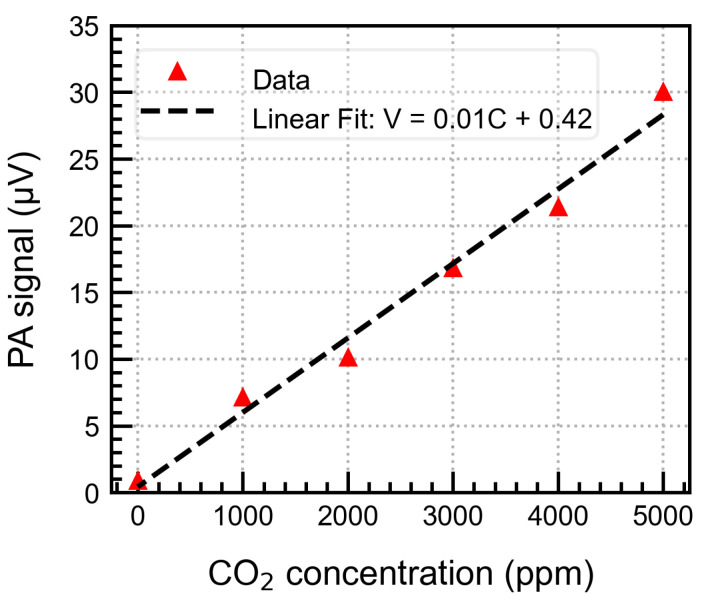
Gas concentration measurements with linear fit (units for the fit parameters are the same as the plotted data units).

Gas measurements were performed to investigate the system’s sensitivity and potential for CO_2_ detection. We varied the gas concentration in steps of 1000 ppm and recorded the PA signal by averaging the data for 200 s for each step. The measurements were performed at 68 Hz, a heater current of 2.5 mA, and a back volume of 0.5 mL. The gas concentration change was performed manually as explained previously. The system exhibited a linear response up to 5000 ppm as expected from theory and validated by a linear fit shown in Figure 14. The standard deviation (σ) per point was less than 0.1 μV. A concentration of 1000 ppm of the gas could be easily detected with higher than ±3σ accuracy with a sensitivity of 6 nV/ppm. The microphone’s sensitivity should be increased to detect even lower gas concentrations.

### 4.6. Allan Variance and Long-Term Stability

**Figure 15:**
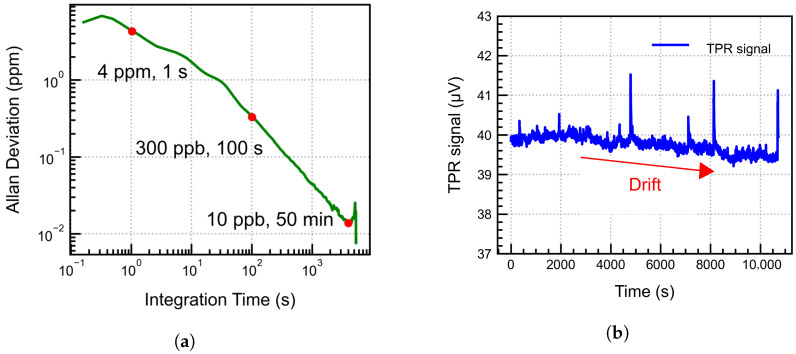
(**a**) Allan Deviation plot for the developed PAS system; (**b**) continuous time PA signal for around 3 h. A slight drift is observed, accompanied by noisy peaks, due to chopper instability.

Two parameters, namely the minimum detection limit (MDL) and the normalised noise equivalent absorption (NNEA), can be used to evaluate the performance of any PAS system. The MDL represents the minimum gas concentration that the system can detect, while the NNEA quantifies the sensor’s performance independently of the laser power, gas line absorption strength, and equivalent noise bandwidth of the lock-in amplifier, thus allowing for the comparison of different PAS systems even when the test conditions are not identical. The NNEA is expressed as [28]

(17) $$\mathrm{NNEA}=\frac{\alpha_{\min}\cdot P}{\sqrt{∆f}}$$

where $\alpha_{\min}$ is the minimum detectable absorption coefficient of the gas absorption line in cm^−1^, *P* is the optical power in W, and ∆f is the detection bandwidth in Hz, usually decided by the lock-in amplifier. The Allan variance plotted in Figure 15a indicates a strong dependence on the 1/f noise as expected. A gas concentration of around 300 ppb can be measured after an integration time of 100 s, and approximately 4 ppm at 1 s. However, the system shows a theoretical MDL of 10 ppb after 50 min.

From the measurements, the system shows a signal of 0.8 μV for 100% N_2_ sealed in the detector cell, which defines the noise level of the system. This corresponds to the detection limit of approximately 130 ppm, given the system sensitivity of 6 nV/ppm. The strong noise level is due to the thermal effects of IR absorption by the optical window and the cell walls. Using these values, the NNEA is calculated to be ∼$6\times 10^{-6}$ W cm^−1^Hz^−1/2^ for our system, which is comparable to the values for a non-resonant system as provided in the literature [29,30,31].

For the long-term stability of the system, the PA signal for 5000 ppm CO_2_ over a period of 2.8 h was measured, and the data is plotted in Figure 15b. The signal exhibits a slight drift, which may be attributed to gas leaking out of the detector cell, thereby compromising system stability over the long term. The leakage rate is calculated around 0.5 ppm/min using the long term data. To avoid this in the future, we plan to use a hermetically sealed system that does not utilise the PCB or other lab-based components. The random, noisy peaks in the signal are due to chopper frequency instability, which were carefully filtered out in the data processing for all measurements performed in this work. In the future, a chopper-free approach using electrical modulation of the laser will be used.

## 5. Conclusions

This work investigates and successfully validates the feasibility of a novel MEMS fluidic microphone for a non-resonant photoacoustic gas sensing application. The fluidic microphone is based on a thermal sensing principle and measures a PA pressure-generated flux via on-chip perforation, unlike commercial capacitive microphones, which detect PA pressure directly. The primary objective is to develop a MEMS acoustic sensor that performs better in the infrasound regime, where non-resonant systems typically excel. The fluidic microphone exhibits an acoustic sensitivity of 32 μV/Pa, which remains constant for $f_{\mathrm{ak}}\leq 20$ Hz. SNR of 50 dB at 1 Pa, 20 Hz is extracted via acoustic measurements. The integration in a PA cell requires a precisely designed dual-volume system (front and back) for optimal performance.

The performance of the fluidic microphone integrated PA cell is explained using a detailed analytical model that includes all the critical frequency behaviours involved in the system. The system exhibits a broad-band frequency response, with the thermal sensor design, perforation geometry, cell radius, and gas volume influencing the system bandwidth and signal amplitude. For experimental analysis, a PA detector cell (18 μL volume) is designed utilising a lateral cavity approach that provides special consideration for IR interaction with the fluidic microphone. PA measurements are performed using a DFB laser operating at approximately 4228 nm, corresponding to a strong absorption line for CO_2_ gas. Firstly, the optimal operation point for the laser is extracted by performing a current sweep for 5000 ppm of CO_2_. Secondly, the optimal modulation frequency for the system, approximately 68 Hz, is determined through frequency sweep measurements. The results are fitted to the analytical model to extract specific fit parameters that account for the thermal diffusion effects and the frequency-independent terms. Gas concentration in 1000 ppm steps is easily resolved with a sensitivity of 6 nV/ppm, with reasonable accuracy. The system shows a linear dependence on the effective volume (a parallel combination of the front and back volumes), as well as the heater temperature. Ultimately, long-term PA measurements are performed, revealing a slight drift in the PA signal, which accounts for the system’s long-term instability due to gas leakage and chopper instability. Allan deviation analysis shows a theoretical limit of detection of 300 ppb in 100 s. However, from the measurements, an offset signal (noise) corresponding to around 130 ppm is measured with $100\%\;N_{2}$ gas, possibly due to the thermal effects arising from the window absorption and wall heating.

In this work, we present the impact of all the relevant design parameters on the performance of the fluidic microphone in PA gas sensing applications. The novel detection principle shows significant potential for robust, miniaturised PA gas sensing. In-house fabrication enables flexible system integration while providing the benefit of tunable performance, which is particularly valuable for implementation in various applications. The sensitivity of the fluidic microphone currently limits the sensitivity of the PA system and will be improved in the future. The cell design will be modified to prevent any gas leakage and to minimise the impact of the window IR absorption on the noise. The effect of variations in gas density, thermal conductivity, and dynamic viscosity resulting from changes in gas concentration and temperature on the performance of the fluidic microphone will be investigated in the future.

## Figures and Tables

**Table 1:** Comparison of various detector types relevant for PAS.

Detector	Detection	Compactness	Sensitivity	Bandwidth
Tuning fork (QEPAS) [3]	resonant	Medium	High	Narrow
Cantilever (CEPAS) [4,5,6]	resonant	Low	Very high	Narrow
Commercial MEMS microphone [10,11]	non-resonant (robust)	High	Medium	Broad (20 Hz–20 kHz)
Fluidic microphone (this work)	non-resonant (robust)	High	Low-Medium	Broad (DC-100 Hz)

**Table 2:** Design parameters used for simulation.

Parameter	Value	Unit
$R_{\mathrm{perf}}$	$8\times 10^{6}$	Pa·s·m^−3^
$A_{\mathrm{perf}}$	$24\times 10^{-9}$	m^2^
$r_{\mathrm{fv}}$	$1.25\times 10^{-3}$	m
$l_{\mathrm{fv}}=l_{\mathrm{abs}}$	$3.75\times 10^{-3}$	m
$V_{\mathrm{fv}}$	18.4$\times 10^{-9}$	m^3^
$C_{\mathrm{fv}}$	$1.3\times 10^{-13}$	m^3^·Pa^−1^
$\tau_{\mathrm{TP}}$	$3.2\times 10^{-3}$	s
ζ	fit parameter	-
$V_{\mathrm{eff}}$	fit parameter	m^3^
*G*	fit parameter	-

## Data Availability

The data presented in this study are available on request from the corresponding author.

## Abbreviations

The following abbreviations are used in this manuscript:

PAS	Photoacoustic Sensing
MEMS	Micro electro-mechanical system
IR	Infrared
PA	Photoacoustic
SNR	Signal-to-noise ratio
NNEA	Normalised noise equivalent absorption
TPR	Right thermopile
CO_2_	Carbon dioxide
N_2_	Nitrogen
